# Expressway traffic flow prediction based on MF-TAN and STSA

**DOI:** 10.1371/journal.pone.0297296

**Published:** 2024-02-22

**Authors:** Xi Zhang, Qiang Ren, Ying Zhang, Chunlian Quan, Shuang Guo, Fangwei Li

**Affiliations:** 1 Chongqing College of Mobile Communication, Chongqing, China; 2 Chongqing Key Laboratory of Public Big Data Security Technology, Chongqing, China; 3 Jungwon University, Incheon, South Korea; Southwest Jiaotong University, CHINA

## Abstract

Highly accurate traffic flow prediction is essential for effectively managing traffic congestion, providing real-time travel advice, and reducing travel costs. However, traditional traffic flow prediction models often fail to fully consider the correlation and periodicity among traffic state data and rely on static network topology graphs. To solve this problem, this paper proposes a expressway traffic flow prediction model based on multi-feature spatial-temporal adaptive periodic fused graph convolutional network (MFSTAPFGCN). First, we make fine preprocessing of the raw data to construct a complete and accurate dataset. Second, by deeply investigating the correlation properties among section speed, traffic flow, and section saturation rate, we incorporate these features into a multi-feature temporal attention mechanism in order to dynamically model the correlation of traffic flow in different time periods. Next, we adopt a spatial-temporal adaptive fusion graph convolutional network to capture the daily cycle similarity and potential spatial-temporal dependence of traffic flow data. Finally, the superiority of the proposed MFSTAPFGCN model over the traditional baseline model is verified through comparative experiments on real Electronic Toll Collection (ETC) gantry transaction data, and the effectiveness of each module is demonstrated through ablation experiments.

## Introduction

As an important transportation channel and transportation hub, expressway has a key role in the convenient travel of people, safe transportation of goods and solid economic development. At the end of 2022, China’s total expressway mileage increased to 177 thousand kilometres, which has grown more than 30% for five consecutive years [1]. At the same time, with the spillover of resources from central cities and the continuous growth of car ownership, the traffic between regions is becoming more frequent, which poses a serious challenge to the carrying capacity of expressways [2]. The contradiction between road supply and demand is becoming more and more prominent, and traffic congestion on the expressways is gradually becoming a normalized phenomenon. Traffic congestion on expressways not only reduces travel efficiency, increases the frequency of traffic accidents, and travel costs, but also worsens environmental pollution problems, such as the inability of vehicles to perform at their best due to congestion, which increases fuel consumption and pollutant emissions, and to a certain extent hinders the realization of the carbon peaking and carbon neutrality goals [3]. Accurate and reliable traffic flow forecasting methods are a key core component of Intelligent Transport Systems (ITS) and the basis for improving the efficiency of expressway operations using ITS systems [4]. Therefore, it is of great significance to research expressway traffic state prediction, timely detect changes in traffic state, and provide a basis for traffic management [5]. The 14th Five-Year Plan for Digital Transportation points out that it is one of the important tasks of digital transportation construction during the 14th Five-Year Plan to focus on promoting the application of ETC on expressways to alleviate traffic congestion, improve operational efficiency, strengthen the monitoring, control and scheduling capabilities of expressways [6]. At present, 27 thousand ETC gantries have been built nationwide, and the number of ETC-installed users exceeds 260 million [7]. The gantry system provides a large amount of ETC transaction data. Therefore, in the context of “strong transportation country”, the carbon peaking and carbon neutrality goals and smart expressway, it is necessary to dig out the complex traffic flow change trend through ETC transaction data, so as to further improve the accuracy of traffic status discrimination and prediction precision.

The traffic state changes with time and space. Because of its own characteristics, the traffic network can be divided into different grid graphs according to other geographical locations [8]. Convolutional Neural Networks (CNN) are effective for the extraction of spatial characteristics of spatial-temporal data and have been widely used in many [9–12]. The literature [13] labeled vehicle speed and vehicle density based on a differential entropy approach, and then used CNN to implement supervised congestion prediction based on traffic meta-parameters (temporal, spatial, date, holiday, special road conditions). The literature [14] uses a CNN model to achieve periodic and random prediction of traffic parameter information using empirical mode decomposition methods. The literature [15] designs a hybrid spatial and temporal feature selection algorithm to reconstruct the traffic speed, then uses CNN to extract the features, and finally captures the temporal characteristics of the data by bidirectional GRU to improve the prediction effect. However, CNNs are unable to fully capture spatial correlations for non-Euclidean spaces, in order to portray the graph structure of the traffic grid, the existing research uses the graph convolution model to gather the node information of the traffic network, and uses its spatial correlation to predict the traffic state of each node as a whole. The literature [16, 17] uses CNN and Recurrent Neural Networks (RNN) to simulate temporal correlation, while Graph Convolution Networks (GCN) are used to simulate spatial correlation. However, GCN cannot assign different weights to its adjacent nodes according to their importance in the convolution process, resulting in the same weight of each node, which has an impact on the accuracy. In recent years, it has been found that the dynamic correlation of spatial-temporal nodes in different scenarios can be calculated by introducing a mechanism to focus on traffic data in the spatial-temporal dimension.

Zhu et al. [18] improved the prediction accuracy by introducing a temporal attention mechanism to adjust the importance of each moment based on the Temporal Graph Convolution Network (T-GCN) [19]. Guo et al. [20] analyzed the recent correlation, daily period correlation and weekly period correlation of traffic flow respectively. Firstly, they processed the temporal attention mechanism of traffic flow, then added the attention mechanism to the spatial graph convolution, and designed a spatial-temporal graph convolution model to predict the change of traffic state. However, the existing methods still do not break through the predefined graph limitations, and the static topology graph established by relying on the existing prior knowledge is missing and insufficient, and cannot be directly related to the actual traffic flow prediction work. Yang et al. [21] designed a Spatial-Temporal Adaptive Fusion Graph Network (STAFGN) for capturing incomplete information on the hidden spatial-temporal dependence of the data, but the correlation and periodicity of the data were not considered.

To solve the above problems, this paper proposes MFSTAPFGCN for expressway traffic flow prediction. Firstly, the section speed and section saturation rate are taken as multivariate variables. In the time dimension, a temporal attention mechanism is introduced to describe the dynamic correlation between section speed and traffic flow, section satiation rate and traffic flow. Then, a Spatial-Temporal Adaptive Period Fusion Graph Convolutional Network (STAPFGCN) is constructed, and the period processing layer captures the daily period similarity of traffic flow data, and adaptively fuses the spatial-temporal graphs of different time periods, and then captures their spatial-temporal correlation in parallel. Finally, comparative experiments and ablation experiments were conducted and validated for the proposed model. The prediction results of the model match well the traffic distribution on the actual road with high accuracy. The main contributions of this work are as follows. (1) From the perspective of time, the dynamic temporal correlation between different traffic variables at the same node is characterized by a multi-feature temporal attention mechanism; (2) The period processing layer is added to the spatial-temporal adaptive fusion graph network, which effectively captures the daily period similarity of traffic flow data; (3) A large number of experiments have been done on the actual data with the model. The experimental results show that MFSTAPFGCN has better performance.

## Related work

Several domestic and foreign researchers have invested in the field of traffic prediction research. The three primary categories of traffic prediction methods include statistical learning models, shallow machine learning, and deep learning. For example, Emami et al. [22] introduced a traffic flow prediction method based on Kalman filtering. Xu et al. [23] presented a methodology combining Autoregressive Integrated Moving Average (ARIMA) and Kalman filter techniques for the prediction of road traffic conditions. Evans et al. [24] conducted road section state prediction using the Random Forest (RF) approach. Through experimentation with varied prediction ranges and training data volumes, their algorithm demonstrated superior performance compared to alternative methods. Lv et al. [25] introduced an advanced spatio-temporal prediction model for the traffic revitalization index utilizing a tree structure. This model comprises key components, namely the spatial convolution module, temporal convolution module, and matrix data fusion module, resulting in improved prediction outcomes. Li et al. [26] presented a spatio-temporal model employing multifactor feature capture. This model integrates various influencing factors that impact cab demand at the spatio-temporal level, distinguishing it from existing baseline approaches. Lv et al. [27] introduced Tree Convolutional Networks (TreeCN), which are designed to capture directional and hierarchical features between nodes. Notably, this approach has demonstrated impressive results, even in scenarios involving small-scale aggregated distributions.

Graph Neural Network (GNN) is a hot research topic in recent years, and it is widely used in processing graph structured data [28], and plays an important role in many fields such as computer vision [29] and natural language processing [30]. Because the traffic network structure is a natural graph structure, many scholars have tried to use GNN to capture the spatial dependence of traffic flow data.

Kong et al. [31] proposed a multi-pattern passenger flow prediction framework based on GCN, which takes into account human mobility knowledge and makes the prediction accuracy become higher. Yu et al. [32] first proposed a combination of Graph Convolution and Gated Causal Convolution for modeling temporal and spatial dependencies. Huang et al. [33] integrated diffusion convolution, the sequence to sequence architecture and scheduled sampling techniques to propose diffusion convolution recurrent neural networks, which captured spatial dependence using bidirectional random wandering on the graph, and temporal dependence using an encoding-decoding framework and scheduled sampling. Zhao et al. [19] proposed T-GCN, which uses GCN to capture spatial dependencies in complex topologies, and uses Gated Recurrent Units (GRU) to learn time dependencies in dynamic traffic data. Wang et al. [34] proposed the Spatial-Temporal Graph Neural Network (STGNN) to fully capture complex spatial-temporal patterns. It uses a learnable location attention mechanism to summarize information from adjacent roads, combines RNN and Transformer to capture local and global temporal correlations, respectively. Each of the above models employs two independent GCN or CNN approaches for modeling spatial correlation, while RNN is used for modeling temporal correlation, which splitting the overall spatial-temporal correlation. Song et al. [17] proposed the Spatial-Temporal Synchronous Graph Convolutional Networks (STSGCN), which uses the spatial-temporal synchronization model to describe the complex local spatial-temporal correlation, and effectively captures the spatial-temporal heterogeneity by setting independent weights for different periods. Li et al. [35] proposed a Spatial-Temporal Fusion Graph Neural Networks (STFGNN) based on the temporal graph. This network uses data-driven methods to supplement the hidden spatial-temporal correlation between nodes, and realizes the learning of long-term correlation of spatial-temporal sequences by stacking spatial-temporal fusion processing modules and gated convolution modules. The above models are based on the static topological graph defined by the physical connection relationship or distance, which is missing and inaccurate, and have no direct connection with the actual traffic flow prediction task itself. In the case that the graph cannot be predefined according to prior knowledge, the prediction accuracy of the above models will be greatly affected.

Wu et al. [36] proposed Graph WaveNet, which uses an adaptive adjacency matrix to supplement the potential spatial dependencies in learning data, and uses a one-dimensional convolution of stacked hole causality to efficiently capture the long-term correlation of traffic flow data. Diao et al. [37] proposed a Dynamic Spatial-Temporal Graph Convolutional Neural Networks model to track the spatial correlation of changes. The dynamic Laplacian matrix estimator is used to capture the change of the Laplacian matrix, introducing tensor decomposition to reduce the complexity, and decomposing the real-time traffic flow data into global components of long-term traffic trends and local components of short-term fluctuations. Zhang et al. [38] proposed the graph convolution framework SLCNN, which introduces a dynamic graph structure learning module that can fully consider the dynamic, real-time, complex relationships and uncertainties in the actual environment.

Wu et al. [39] proposed MTGNN, which analyzes multivariate time series from the perspective of a graph for the first time, uses a graph structure learning module to learn potential spatial dependencies between data in end-to-end training, and proposes a joint framework to learn multivariate time series without predefined graph structure in a graph structure, which solves the limitations of existing models when graph structure is unknown or missing to some extent. Guo et al. [40] proposed a novel Dynamic Graph Convolutional Network (DGCN), which introduced a latent network to extract spatial-temporal features for adaptively constructing dynamic road network graph adjacency matrices. Bai et al. [41] proposed an Adaptive Graph Convolutional Recurrent Network (AGCRN) without using a predefined graph, and enhanced the graph convolutional network by using a node adaptive parameter learning module that captures node-specific patterns and an adaptive graph generation module that automatically infers mutual data between different traffic flow time series.

GCN has natural advantages in non-Euclidean data such as traffic networks. Models such as STGCN and T-GCN use GCN to capture spatial correlation, and use RNN and CNN to capture temporal correlation. However, these methods are based on known graph structures and cannot be used for uncertain or dynamically changing graph structures. GraphWaveNet, ASTGCN, GCNN, SLCNN, MTGNN, AGCRN, and DGCN use dynamic graph structures to effectively capture potential data associations. But such models use separate components to capture the temporal and spatial correlation of traffic flow data, splitting the overall spatial-temporal correlation, and not fully considering the spatial-temporal correlation between adjacent nodes at different times. STSGCN is based on the synergistic processing of potential spatial-temporal correlations in traffic flow through time blocks, but it does not evenly process the global and heterogeneous features of traffic flow, resulting in the weakening of temporal correlations between adjacent time periods. In addition, the spatial-temporal correlation of functionally similar road segments, as well as the periodic correlation of traffic flow data and the influence of special events, are not considered, resulting in their inapplicability to the dynamic graph structure.

## Preliminaries

In order to represent the spatial topological relationship of each ETC gantry in the expressway road network, the expressway road network is represented as a graph *G* = (*V*, *E*, *A*), where |*V*| = {*Node*_1_, ⋯*Node*_*n*_} is the set of all ETC gantries on the expressway, *N* denotes the number of ETC gantries; *E* denotes the set of interconnecting edges between ETC gantries. *A* ∈ *R*^*N*×*N*^ means the spatial adjacency matrix of the gantry, and *A*_*ij*_ is 1 if there is a connection between *Node*_*i*_ and *Node*_*j*_, otherwise, it is 0. Traffic flow, section speed and section saturation rate can all be used as attribute features of a node. *X*^*t*^ ∈ *R*^*N*×*d*^ is defined as the value taken by a node at time *t*, and *d* denotes the dimension of traffic flow, section speed and section saturation rate. Expressway traffic flow prediction can be described as learning a mapping function *f* based on the traffic flow at *T* moments in history to predict the zone speed at *T*′ moments in the future.

$$\begin{array}{c} \left[\left(X^{\left(t-T+1\right)},\cdots ,X^{t}\right);G\right]\overset{f}{\to }\left[\left(X^{\left(t+1\right)},\cdots ,X^{t+T^{\prime }}\right);G\right] \end{array}$$ (1)

## Methodology

**Fig 1:**
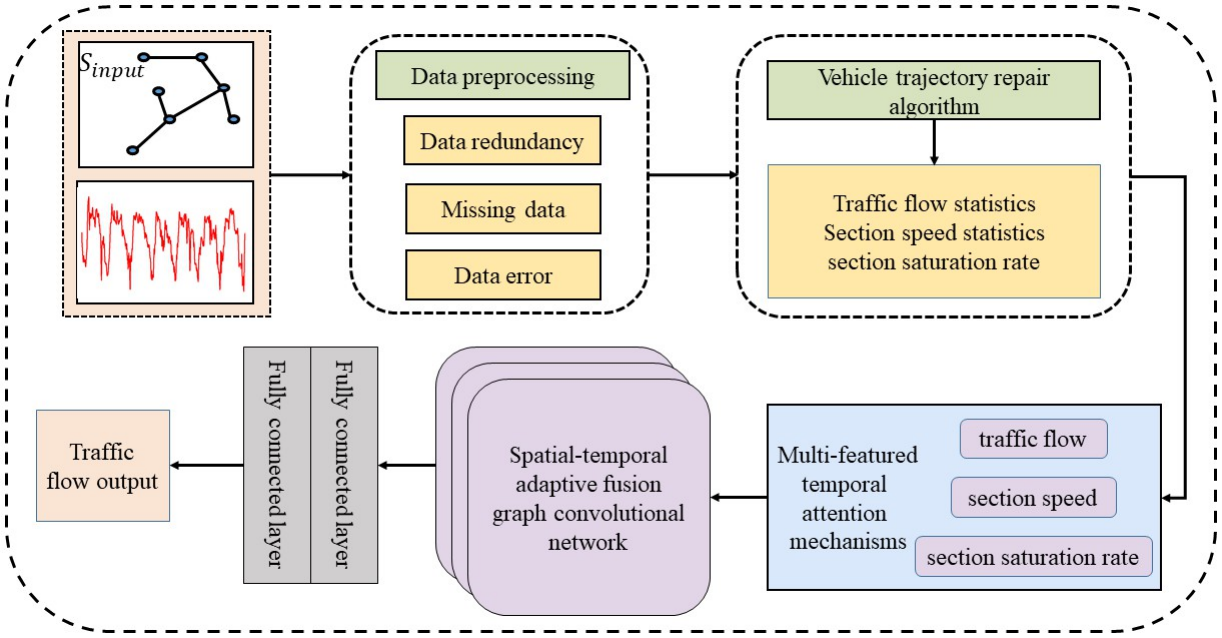
The overall framework of MFSTAPFGCN.

The general framework of the proposed MFSTAPFGCN model is shown in Fig 1. It consists of three main parts. The first part is to process the redundant and missing data in the ETC gantry transaction data in a relevant way, and then use the vehicle trajectory repair algorithm to repair the erroneous data, and then construct the traffic flow data. The second part constructs a multi-featured temporal attention mechanism, and uses the correlation functions of section speed and section saturation rate with traffic flow to fuse and calculate the process state importance at each moment, and enhance the model’s ability to portray the feature correlation on the time sequence. The third part constructs a Spatial-Temporal Adaptive Fusion Graph Convolutional Network (STFAGCN), It consists of four modules, firstly a cycle processing module based on splicing, dimension expanding, and diffusion convolution operations to model the similarities of daily cycles of traffic flow data; then a spatial-temporal adaptive fusion graph construction module to realize the effective fusion of temporal and spatial; and finally, a spatial-temporal adaptive fusion graph module and a gated convolution module to adaptively extract the long-time spatial-temporal correlations.

### Data preprocessing

Due to the influence of uncontrollable random factors such as equipment abnormality, wireless crosstalk and bad weather, ETC transaction data will produce the following three types of abnormal problems in the collection process [42]. (1) Data redundancy: There will be duplication between multiple data, which will lead to waste of storage space, complex modification and potential data inconsistency. (2) Missing data: There will be some key segments such as date, time, vehicle type, vehicle model, etc. in and out of the toll station missing, resulting in the inability to effectively collect data, thus causing unreliable output, and there are generally two methods to deal with the missing problem: deletion and interpolation. (3) Data error: data that is inconsistent with normal traffic rules, such as pass numbers and incorrect gantry numbers. These abnormal data reduce the application value of ETC data mining. Firstly, redundant data and error data should be eliminated, and missing data should be repaired to reduce the impact on the model and improve the reliability of the prediction.

Vehicle trajectory repair algorithm [42]: Build the running path of each car according to chronological order. By searching the ETC gantry on the vehicle running path and the adjacent ETC gantry on the path, the topological relationship between the adjacent ETC gantry in the expressway *G* is determined. If so, the travel time and speed can be calculated directly. If not, a segment search is carried out according to the two gantries and inserts the running path of the car. The average speed of cars in each lane is calculated, which is used as the speed of each lane, and the driving time between lanes is obtained by the distance between lanes. In this way, the running route of the car can be restored.

### Multi-feature temporal attention mechanism

From the temporal perspective, there is a certain correlation between the traffic flows of ETC gantries in the expressway road network at different time points, and this correlation changes with the change in road network conditions. Guo et al. [20] proposed a method for traffic flow prediction based on an attention mechanism that assigns different importance to the traffic flow of the road network at different moments in time, and their temporal attention mechanism in the time domain is shown as follows:

$$\begin{array}{c} E=V_{e}\sigma \left({\left(U_{1}X^{t}\right)}^{T}U_{2}\left(U_{3}X^{t}\right)+b_{e}\right) \end{array}$$ (2)

where *V*_*e*_, *b*_*e*_ ∈ *R*^*T*×*T*^, *V*_*e*_, *b*_*e*_ ∈ *R*^*N*^, *U*_3_ ∈ *R*^1^ are the parameters to be learned.

Each ETC gantry in the expressway network records different time series, such as traffic flow, section speed, and section saturation rate. The three variables of the same ETC gantries are correlated among different moments, and the correlation of the section speed and section saturation rate to the traffic flow is calculated separately with the help of cosine similarity, and the formulas are:

$$\begin{array}{c} \text{COS}\left(a,b\right)=\frac{a\cdot b}{∥a∥\cdot ∥b∥} \end{array}$$ (3)

$$\begin{array}{c} M_{1}=\left[\text{cos}\left(x_{f}^{t},x_{s}^{t}\right)\right] \end{array}$$ (4)

$$\begin{array}{c} M_{2}=\left[\text{cos}\left(x_{f}^{t},x_{o}^{t}\right)\right] \end{array}$$ (5)

$x_{f}^{t},x_{s}^{t},x_{o}^{t}$ mean the traffic flow, section speed measured and section saturation rate by *N* ETC gantries in time period *t* respectively, *M*_1_ is the similarity matrix of traffic flow and section speed, and *M*_2_ is the similarity matrix of traffic flow and section saturation rate.

**Fig 2:**
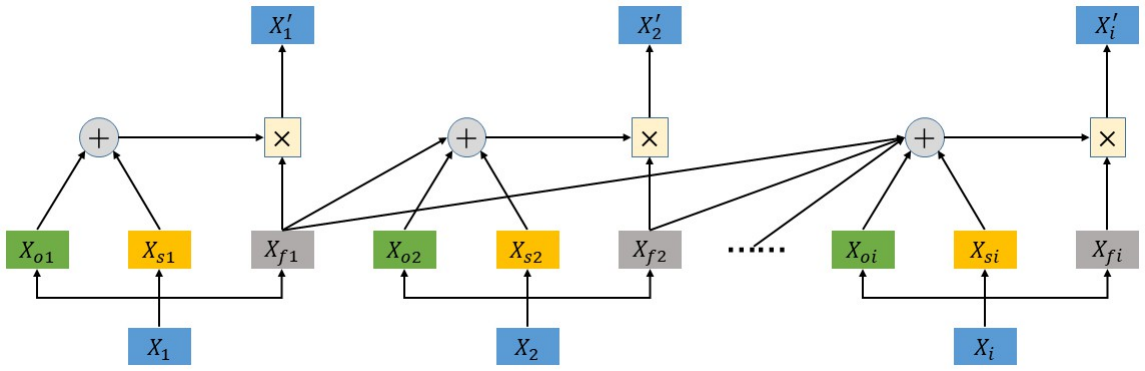
Multi-feature temporal attention mechanism.

The multi-feature temporal attention mechanism is used to assign different importance to the traffic state data of the road network at different moments. The difference with the literature [20] is that the multi-feature temporal attention mechanism considers the correlation in both time and variable dimensions. The structure of the multi-feature temporal attention mechanism is shown in Fig 2. The blue part *X*_1_ is the input data of the first moment of a certain ETC gantry, containing the multi-dimensional parameters of section saturation rate *X*_*o*1_, section speed *X*_*s*1_ and traffic flow *X*_*f*1_. The multidimensional data of the ETC ganZtry measured once in 15 minutes are parameterized, and finally the new traffic flow $X_{1}^{\prime },\cdots ,X_{i}^{\prime }$ is obtained. The specific parameter fusion process is as follows:

When calculating the first moment $X_{1}^{\prime }$, the influence of section saturation rate and section speed on traffic flow is calculated by adding green section saturation rate *X*_*o*1_ and yellow section speed *X*_*s*1_ and multiplying with gray traffic flow *X*_*f*_1. When calculating $X_{i}^{\prime }$, the traffic flow of the previous *i* time steps is considered to have an impact on the traffic flow at the current *i* moment, which is obtained by adding the traffic flow at the previous *i* − 1 moment and the current moment’s section saturation rate and section speed and multiplying them with the current traffic flow. Finally, using the activation function to make a projection into a new function space, this is done for each input data, and then using Softmax $E_{i,j}^{\prime }$ is calculated. The calculation formula is as follows:

$$\begin{array}{c} E=V_{e}\sigma \left({\left(U_{1}X^{t}\right)}^{T}\left(U_{2}+M_{1}Q_{1}+M_{2}Q_{2}\right)\left(U_{3}X^{t}\right)+b_{e}\right) \end{array}$$ (6)

$$\begin{array}{c} E_{i,j}^{\prime }=\frac{\text{exp}(E_{i,j})}{\sum_{j=1}^{T_{h}}\text{exp}\left(E_{i,j}\right)} \end{array}$$ (7)

*E*_*i*,*j*_ represents the correlation of the traffic state data of the road network at moment *i*, *j*. $E_{i,j}^{\prime }$ is the correlation of the traffic state data of the road network nodes at moment *i*, *j* after normalization. The multi-feature time attention matrix *E* is determined by the input, and as the input changes, *E* also changes with it, so that the multi-feature time attention mechanism can dynamically obtain multiple information correlations on the time domain of traffic data. Similarly, a Softmax normalization of the elements on *E* is required. The normalized multi-feature time attention matrix $E^{\prime }$ is directly applied to the input to obtain the traffic flow updated by the multi-feature temporal attention mechanism.

### Spatial-temporal adaptive period fusion graph convolutional network

**Fig 3:**
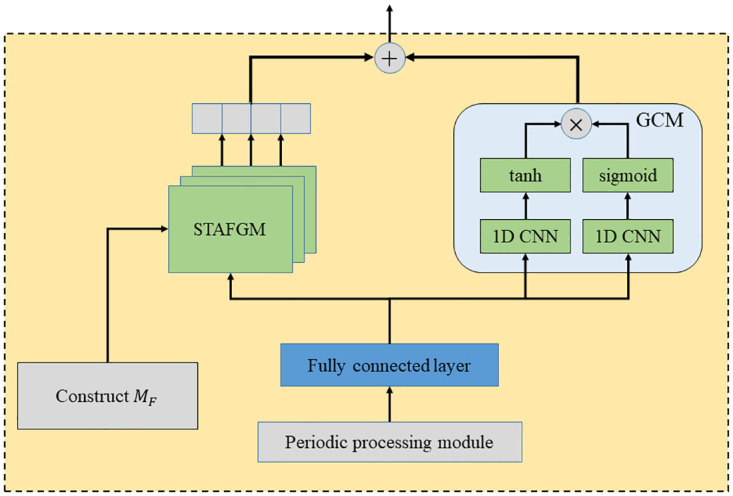
Spatial-temporal adaptive period fusion graph convolutional network.

As shown in Fig 3, the STAPFGCN consists of four modules, including the Period Processing Module (PPM), Spatial-Temporal Adaptive Fusion Graph Construct Module (STAFGCM), Spatial-Temporal Adaptive Fusion Graph Module (STAFGM) and Gated Convolution Module (GCM). Firstly, the PPM based on splicing, dimensional expansion, and diffusion convolution operations is designed, and the daily period similarity of traffic flow data is modeled. Second, the temporal and spatial fusion adjacency matrix *M*_*F*_ is constructed using STAFGCM to achieve an effective fusion of temporal and spatial. *M*_*F*_ contains the temporal adjacency matrix *A*_*TG*_, spatial adjacency matrix *A*_*SG*_, and temporal connectivity graph matrix *A*_*TC*_ computed by fast-DTW [35]. Then, a STAFGM based on fused adaptive convolutional layers and stacked gated multiplication layers with maximum pooling action is proposed. By the end-to-end supervised method, the adjacency matrix is continuously learned in the adaptive convolution layer to construct the adaptive fused adjacency matrix ${\tilde{M}}_{F}$. Finally, the GCM uses a highly scalable grid method to extract long-term spatial-temporal correlations, stacking *k* STFAGN layers to capture potential spatial-temporal dependencies.

#### Period processing module

**Fig 4:**
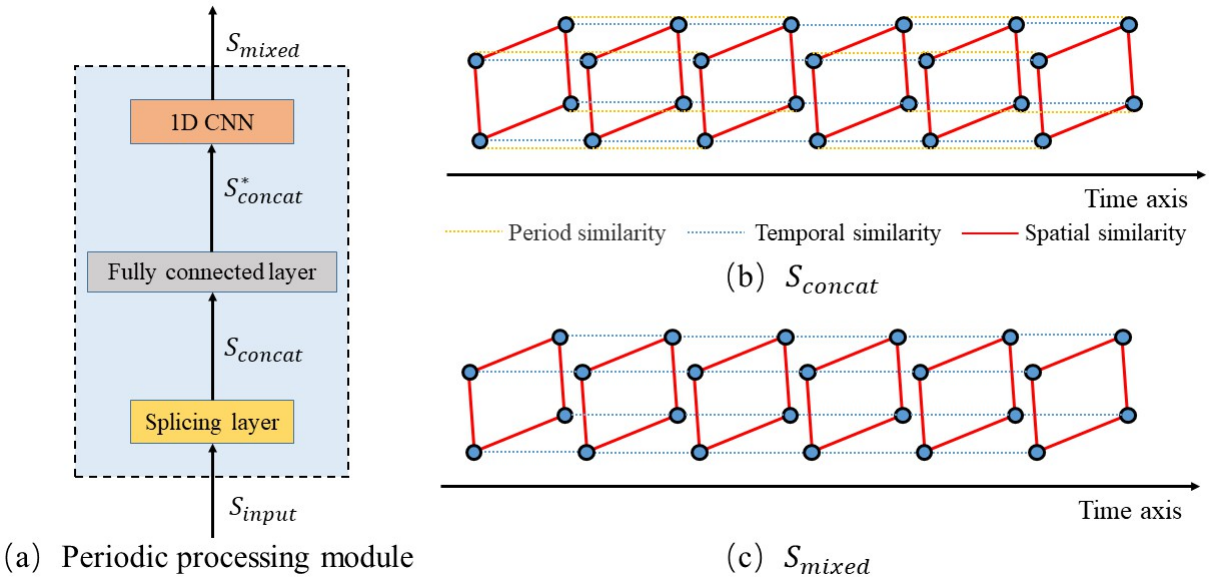
Period processing module.

The form of the period processing module is shown in Fig 4(a), where the *N*_*P*_ strips of spatial-temporal sequential traffic flow *S*_*input*_ input model with the periodical association after updated by the multi-feature temporal attention mechanism are cross-spliced by the period processing module to obtain *S*_*concat*_ in the form shown in Fig 4(b), where the yellow line represents the interconnection between the nodes and their predecessors or successors on the period axis. *S*_*concat*_ is expanded with feature channels by a fully connected layer, and finally, by using a 1D CNN to process $S_{concat}^{*}$, the output size of the convolutional layer is calculated as follows:

$$\begin{array}{c} \text{Outputshape}=\frac{\text{Inputshape}-\text{Filtershape}+2\text{Pad}}{\text{Stride}}+1 \end{array}$$ (8)

Where *Inputshape* = *N*_*p*_ × *T*, *Filtershape* = *N*_*p*_, *Pad* = 0, *Stride* = *N*_*p*_, the output of the convolution layer is the output of the period fusion processing layer, and the length *T* contains the mixed spatial-temporal sequence *S*_*mixed*_ with periodic features, in the form of Fig 4(c).

#### Spatial-temporal adaptive fusion graph construct module

The spatial-temporal fusion adjacency matrix *M*_*F*_ ∈ *R*^*KN*×*KN*^ consists of the fast-DTW computed temporal adjacency matrix *A*_*TG*_ ∈ *R*^*N*×*N*^, the spatial adjacency matrix *A*_*SG*_ ∈ *R*^*N*×*N*^ and the temporal connectivity graph matrix *A*_*TC*_ ∈ *R*^*N*×*N*^. STFGNN handles temporal by introducing the data-driven temporal adjacency matrix *A*_*TG*_ ∈ *R*^*N*×*N*^ non-neighborhood similarity between nodes with highly similar patterns. Given two time series *X* = (*x*_1_, *x*_2_, ⋯, *x*_*n*_) and *Y* = (*Y*_1_, *Y*_2_, ⋯, *Y*_*m*_), a distance matrix *M*_*n*×*m*_ can be introduced with *M*_*i*,*j*_ = |*x*_*i*_ − *y*_*j*_|. Then the cost matrix *M*_*c*_ can be defined by the following equation:

$$\begin{array}{c} M_{c}\left(i,j\right)=M_{i,j}+\text{min}\left(M_{c}\left(i,j-1\right),M_{c}\left(i-1,j\right),M_{c}\left(i,j\right)\right) \end{array}$$ (9)

After several iterations of *i* and *j*, $M_{c}{\left(i,j\right)}^{\frac{1}{2}}$ is the best similarity between time series *X* and time series *Y*. DTW [43] obtained twisted paths by matching *x*_*i*_ and *y*_*j*_ based on dynamic programming, and *w*_*ξ*_ denotes the matching of *x*_*i*_ and *y*_*j*_.

$$\begin{array}{c} \Omega =(w_{1},w_{2},\cdots ,w_{\xi }) \end{array}$$ (10)

where *max*(*n*, *m*) ≤ *ξ* ≤ *n* + *m*. It follows that the algorithmic complexity of fast-DTW is *O*(*n*^2^) can be reduced to *O*(*Tn*) by limiting the search length *T* [35]:

$$\begin{array}{c} w_{k}=\left(i,j\right),|i-j|\leq T \end{array}$$ (11)

**Fig 5:**
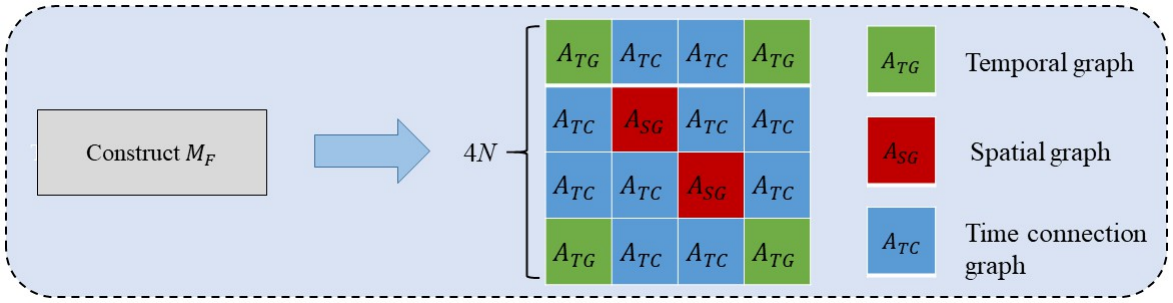
Spatio-temporal adaptive fusion graph construct.

Adding temporal trend similarity to fast-DTW for constructing temporal adjacency matrix. In the temporal adjacency matrix, the *K* nodes that are most similar to node *N* in terms of temporal patterns are considered to be interconnected, and the value of *K* in STFGNN is fixed as *K* = *sparsity* × *N*, where *sparsity* is a hyperparameter given in advance based on experience. However, the lack of complete connectivity prevents the model from effectively learning the nonproximate similarities between nodes, which in turn affects the prediction accuracy of the model. Using Algorithm1 in the literature [35] to calculate the temporal graph, the hyperparameter *K* is the degree of nodes in the temporal graph, which determines the sparsity of the temporal graph and has a large impact on the prediction accuracy of the model, and hyperparameter optimization experiments need to be conducted for the value of *K*. *A*_*TG*_ means the correlation between a node and its own predecessor or successor nodes on the time axis. At each node *l* ∈ {1, 2, ⋯, *N*}, *M*_*F*(*ij*)_ = 1 when *i* = *t* × *N* + *l* and *j* = (*t* + 1) × *N* + *l*, where *t* is the current time step. Using matrix multiplication with *M*_*F*_, the spatial correlation, temporal correlation, and autocorrelation from the nearest time step are integrated for each node. Finally, the spatial-temporal adaptive fusion adjacency matrix ${\tilde{M}}_{F}$ is constructed. The spatial-temporal adaptive fusion graph construction module is shown in Fig 5.

#### Spatial-temporal adaptive fusion graph module

**Fig 6:**
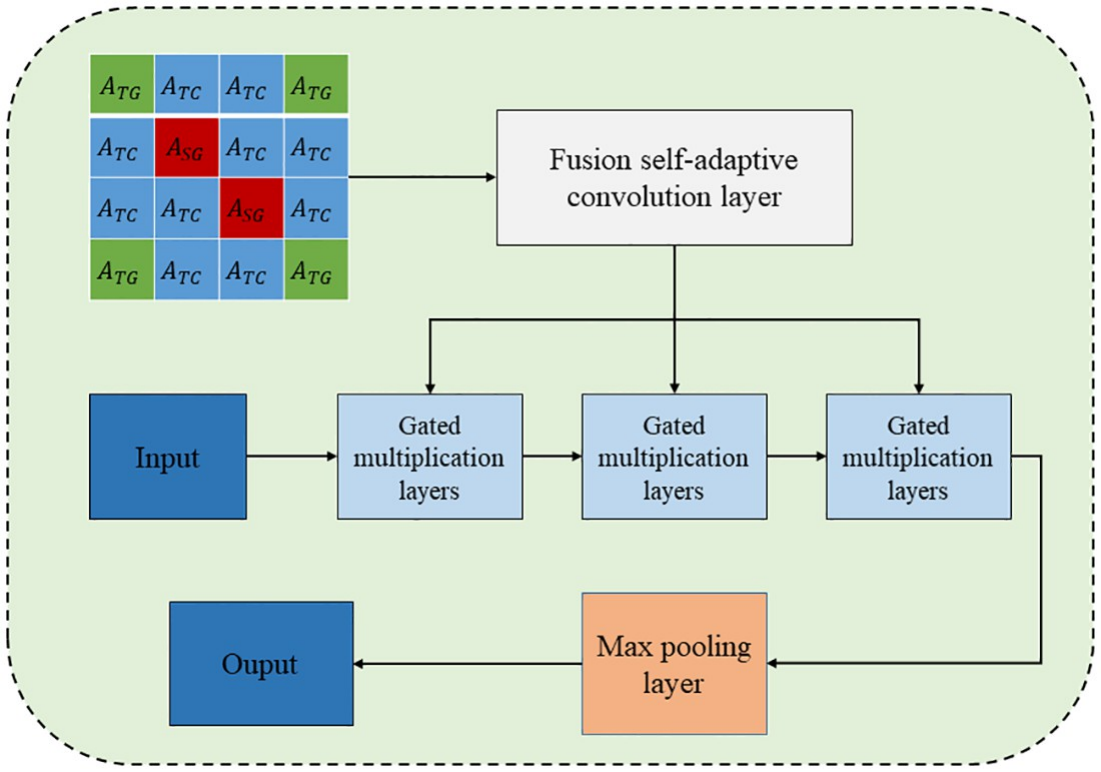
Spatial-temporal adaptive fusion graph module.

Fig 6 shows the STAFGM. The STAFGM module consists of fused adaptive convolution layers with stacked gated multiplication layers and a maximum pooling layer connected to ReZero [44]. The input of each fusion self-adaptive convolution layer is the output of the PPM or the output of the previous fusion self-adaptive convolution layer. In order to be able to complete the incomplete connection of graphs adaptively, Yang et al. [21] introduced a spatial-temporal adaptive fusion graph module in the STFGNN. Compared with STFGNN, the spatial-temporal adaptive fusion graph module completes the incomplete spatial-temporal adjacency matrix by constructing the fused adjacency matrix of spatial-temporal relations.

Fusion Self-Adaptive Convolution layer: based on spatial-temporal fusion adjacency matrix and diffusion adaptive convolution, a new fusion adaptive convolution (FSAC) [21] algorithm is designed to realize the adaptive fusion of adjacency matrix ${\tilde{M}}_{F}$.

${\tilde{M}}_{F}=softmax\left(ReLu\left(M_{i}M_{j}^{T}\right)\right)$ , where *M*_*i*_ and *M*_*j*_ are the spatial-temporal fused node embeddings of the source and target nodes, which are learnable parameters, and the ReLU activation function is used to prevent the occurrence of overfitting. Define *S*_*f*_ = *M*_*F*_/*rowsum*(*M*_*F*_) and $S_{b}=M_{F}^{T}/rowsum\left(M_{F}^{T}\right)$. Finally, the convolutional layer of the graph with ${\tilde{M}}_{F}$ can be summarized as:

$$\begin{array}{c} ϒ=\sum_{K=0}^{K}{\tilde{M}}_{F}^{k}{\text{XW}}_{F}^{k}+S_{b}^{k}{\text{XW}}_{f}^{k}+S_{f}^{b}{\text{XW}}_{b}^{k} \end{array}$$ (12)

Gated multiplication layer: Each node in *H*^0^ is synchronized with complex correlations of spatially adjacent nodes, non-neighboring similar nodes, and adjacent temporal nodes by Lth matrix multiplication with ${\tilde{M}}_{F}$. The graph convolution module also introduces the nonlinear activation property of the gating unit:

$$\begin{array}{c} H^{l+1}=\left({\tilde{M}}_{F}H^{l}W_{1}+b_{1}\right)⊙\sigma \left({\tilde{M}}_{F}H^{l}W_{2}+b_{2}\right) \end{array}$$ (13)

where *H*^*l*^ represents the lth hidden state, ${\tilde{M}}_{F}$ is the spatial-temporal adaptive fusion graph, *W*_1_, *W*_2_ and *b*_1_, *b*_2_ are the parameters of the GLU module, ⊙ the Hadamard product, and *σ* represents the sigmoid function. The spatial-temporal adaptive fusion graph module aggregates complex nonlocal spatial dependencies by stacking *L* graph convolutions in combination with ReZero connections [45]. To reduce the complexity, the maximum pooling layer output *H*^*M*^ = *maxpool*(*H*^1^, ⋯, *H*^*L*^) keeps only the intermediate time steps, so the spatial-temporal adaptive fusion graph module output *H*^0^ is as follows:

$$\begin{array}{c} H^{0}=H^{M}[[\frac{K}{2}]:[\frac{K}{2}+1],:,:] \end{array}$$ (14)

#### Gated convolution module

Gated TCNs with different dilation factors *k* can learn complex temporal correlations [36]. the GCM uses a larger dilation rate and two parallel 1D dilation convolutions to form the GCM, which can effectively obtain the long-term temporal dependence of the spatial-temporal sequence. The output of the gated convolution module is calculated by the following:

$$\begin{array}{c} Z=\phi (\theta_{1}✱X+a)⊙\sigma (\theta_{2}✱X+b) \end{array}$$ (15)

where *θ* represents the tanh function, *σ* represents the sigmoid function, and *θ*_1_ and *θ*_2_ are two independent 1D dilation convolutions with a *K* − 1 expansion rate. It could enlarge the receptive field along the time axis thus strengthening model performance for extracting spatial-temporal dependence.

## Experiments

The experimental setup for this study was configured as follows: Our model was trained on a computer running the Windows 11 operating system, equipped with an AMD Ryzen 7 5800H eight-core processor (with a base frequency of 3.2GHz) and an NVIDIA GeForce RTX 4060 Laptop GPU. The system was supported by 16GB of memory. We utilized the Anaconda software distribution and JetBrains’ Python development tool.

### Data

**Fig 7:**
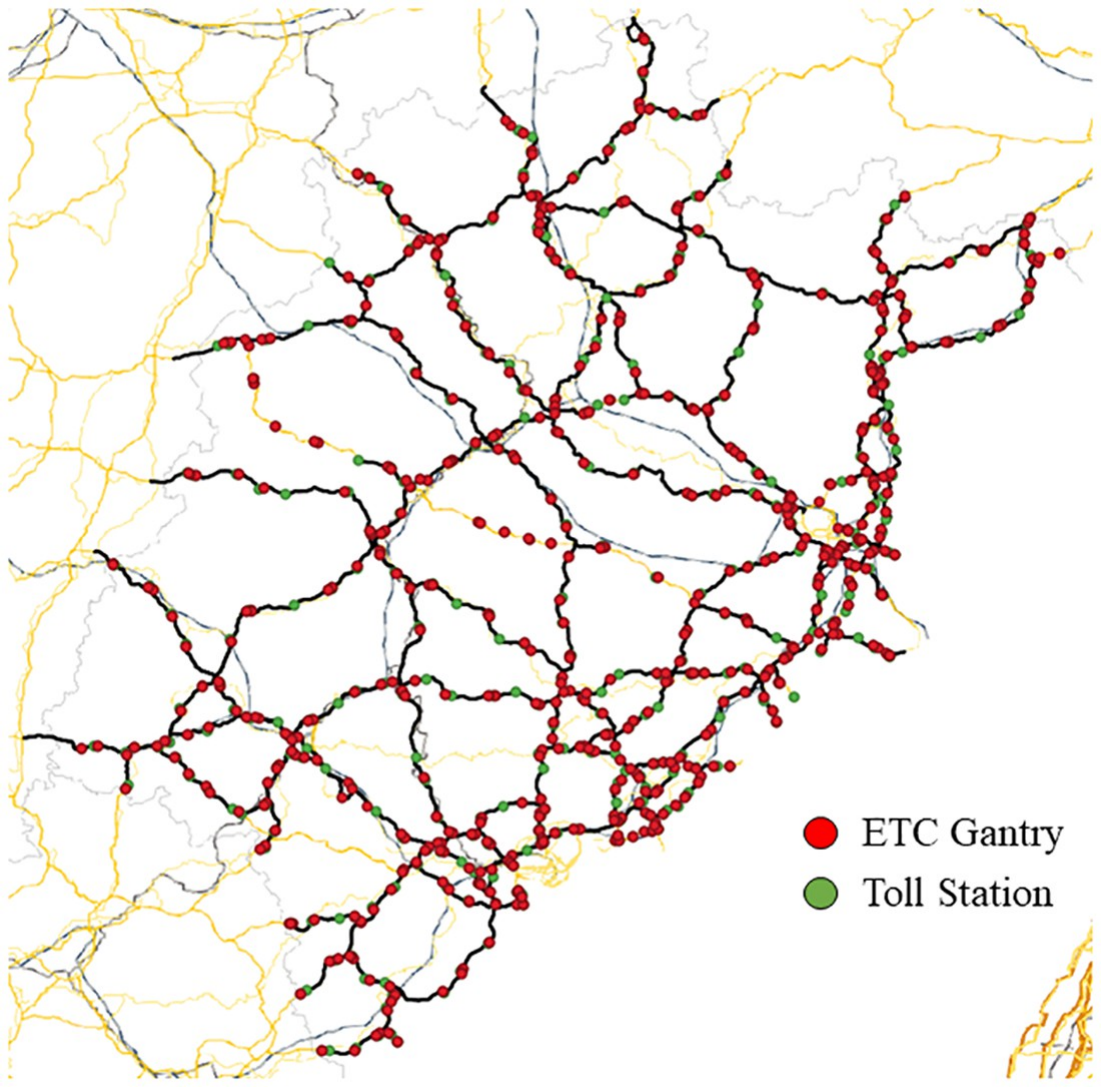
Map of the ETC gantry system setup.

The data used in this paper are mainly divided into two types of data, one is the ETC gantry transaction data of a province from May 2021, the main attributes are shown in Table 1, the layout of the gantry is shown in Fig 7. Two is the road network topology data, which contains each ETC gantry, the connection relationship between ETC gantries and the actual distance. The traffic flow data of each ETC gantry are counted and aggregated into a 15-minute window. The data are normalized by removing the mean and scaling to unit variance:

$$\begin{array}{c} X^{\prime }=\frac{X-\text{mean}(X)}{\text{std}(X)} \end{array}$$ (16)

where *mean*(*X*) and *std*(*X*) are the mean and standard deviation of the traffic flow data, respectively.

**Table 1:** Partial attribute table of ETC gantry transaction data (*** indicates that the data was desensitized).

Attribute name	Examples	Attribute name	Examples
Trade ID	3408119***2698	OBU SN	09351***32YT9
Trade Time	2021/5/1 13:27:51	Vehicle Class	1
Flag ID	340A27	Enter Time	2021/5/1 12:21:39
User Type	0	Enter Station	3506
Flag Type	0	OBU ID	96E***45C

### Evaluation metrics and experiment setup

To evaluate the prediction performance of different models, the experiments use Mean Absolute Error (MAE), Mean Absolute Percentage Error (MAPE), and Root Mean Square Error (RMSE) as evaluation metrics. And Hubor Loss was used as the loss function. Compared with the common loss function, Hubor Loss is more robust against outliers, and according to Eq (20), the squared error is used when the error is less than *δ*, and the linear error is used when the error is greater than *δ*, and the training speed is improved.

$$\begin{array}{c} \text{MAE}=\frac{1}{N}\sum_{i=1}^{N}|Y_{i}-{\hat{Y}}_{i}| \end{array}$$ (17)

$$\begin{array}{c} \text{MAPE}=\frac{1}{N}\sum_{i=1}^{N}|\frac{Y_{i}-{\hat{Y}}_{i}}{Y_{i}}| \end{array}$$ (18)

$$\begin{array}{c} \text{RMSE}=\sqrt{\frac{1}{N}\sum_{i=1}^{N}{\left(Y_{i}-{\hat{Y}}_{i}\right)}^{2}} \end{array}$$ (19)

$$\begin{array}{c} h\left(\hat{Y},Y\right)=\{\begin{array}{cc} \frac{1}{2}{\left(\hat{Y}-Y\right)}^{2} & |\hat{Y}-Y|\leq \delta \\ \delta |\hat{Y}-Y|-\frac{1}{2}\delta^{2} & |\hat{Y}-Y|>\delta \end{array} \end{array}$$ (20)

where *Y* is the actual traffic flow, $\hat{Y}$ is the predicted traffic flow, *N* is the number of ETC gantries, and *δ* is the hyperparameter.

The data set is split into the training set, validation set, and test set according to the ratio of 6:2:2, and all experiments are repeated ten times to take the average. The hyperparameters involved are configured as follows: *N*_*p*_ is 3, the sparsity of the temporal adjacency graph *A*_*TG*_ is 0.01, the number of layers of STAFGL for this model is 3, each STAFGL consists of 9 STAFGM and 1 gated convolution module with a dilation rate of 3, the filter used for all convolutions is 64, the learning rate is 0.001 using the Adam optimizer, the threshold *δ* in the loss function is 1, the batch size is 32, and the training epoch is 100. We use the last 12 consecutive time steps to predict the traffic flow for the next hour.

The sparsity of the temporal graph *A*_*TG*_ has a large impact on the prediction accuracy of the model. Specifically, a denser temporal graph contains more comprehensive potentially spatially relevant information, which helps the model learn the complex spatial-temporal dependencies at the overall level of the large-scale road network structure. On the other hand, a too-densely connected temporal graph weakens the influence weights of the more valuable nodes, making the prediction results more generalized and smooth, and more prone to overfitting. To ensure that the model can learn more adequate and necessary non-neighboring potential associations, the following optimization-seeking experiments are set up for the hyperparameter *K*.

**Fig 8:**
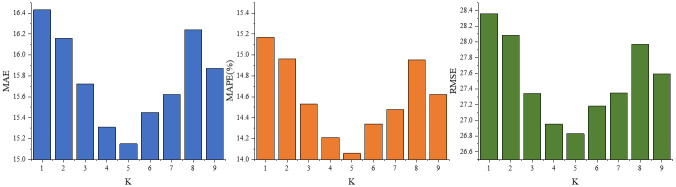
Optimization-seeking experiment with hyperparameters *K*.

The results of the optimization search experiment are shown in Fig 8. When *K* = 5, the model has the best prediction performance, and the temporal graph generated by the five nodes connecting the nodes with the most similar temporal patterns to theirs is the local optimal temporal graph corresponding to the data set.

### Baseline models

In order to fully validate the prediction performance of MFSTAPFGCN, a fair comparison is made with the following eight representative traffic forecasting models.

FC-LSTM [46]: long short-term memory neural network, using forgetting gate, input gate, and output gate structures to process different state information separately, modeling the time series long time dependence.

DCRNN [33]: diffusion convolution recurrent neural network, using diffusion graph convolution to encode spatial information, using seq2seq to encode temporal information, and extending graph convolution to directed graphs.

STGCN [32]: spatial-temporal graph convolution network that captures the spatial and temporal correlation of the data using ChebNet and 2D convolutional networks, respectively.

ASTGCN [20]: spatial-temporal graph convolutional network based on attention mechanism using spatial attention mechanism to deal with dynamic spatial correlation and temporal attention mechanism to deal with dynamic temporal correlation, and weighted fusion of three independent components to model the multi-scale periodicity of traffic data.

Graph WaveNet [36]: Graph WaveNet uses an adaptive adjacency matrix to complement the hidden spatial dependencies in the learning data and combines graph convolution, null causal one-dimensional convolution to capture long-term temporal relationships at low cost.

STSGCN [17]: spatial-temporal synchronized graph convolutional network uses localized spatial-temporal subgraph modules to synchronously model localized spatial-temporal correlations in spatial-temporal networks.

STFGNN [35]: spatial-temporal fusion graph neural network introduces data-driven temporal graphs and models spatial correlation, non-neighborhood similarity, and temporal correlation in spatial-temporal networks by parallel stacking of spatial-temporal fusion layers and gated expansion convolutional fusion.

STFAGN [21]: combines fused convolutional layers with a new adaptive dependency matrix through end-to-end training to capture hidden spatial-temporal dependencies on the data to complete incomplete information.

### Experimental results and analysis

**Fig 9:**
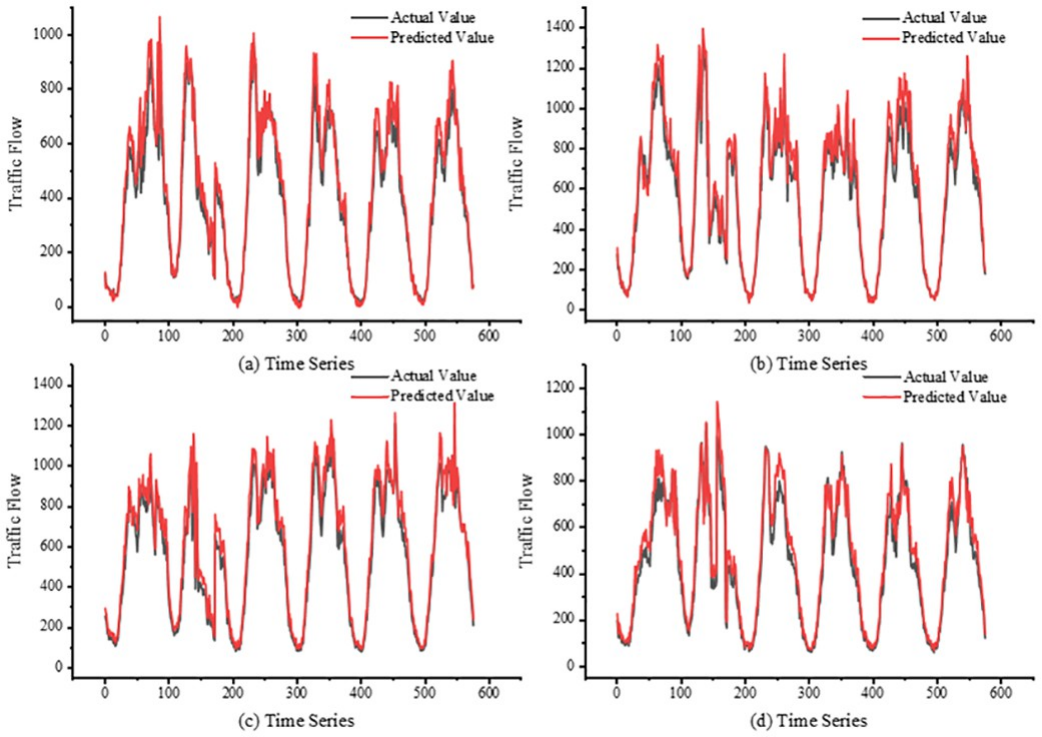
Visualization results for 15-min. (a)Section a (b)Section b (c)Section c (d)Section d.

The experiment uses 12 historical data samples to predict the future 15-min, 30-min, 45-min, 60-min traffic flow. Fig 9 shows the results of the visualization of the predicted, they are four randomly selected sections. The black color in the figure indicates the predicted traffic flow and the red color indicates the actual traffic flow. The prediction accuracy decreases in the road sections where the traffic flow changes in a complicated way, but the model can capture a similar trend when the traffic flow changes suddenly.

Table 2 shows the evaluation metrics for each model prediction result. Since the FC-LSTM model only considers temporal correlation and does not fully exploit the spatial correlation of traffic flow data, the prediction performance accuracy is much lower than other graph convolutional-based models with spatial correlation, which cannot meet the large-scale traffic flow prediction tasks. Graph WaveNet uses an adaptive adjacency matrix to complement the hidden spatial dependence in the learning data, because it cannot simultaneously superimpose on time and spatial, and therefore has the worst prediction performance among the graph convolutional neural network-based approaches. DCRNN, STGCN, and ASTGCN use separate components to deal with temporal and spatial correlations in traffic flow data separately, splitting the overall spatial-temporal correlations. STSGCN uses local spatial-temporal graphs to model spatial-temporal correlations, local heterogeneity simultaneously and has better prediction results. However, the spatial-temporal correlation and heterogeneity properties are not balanced, and the spatial-temporal graph weights are independent of each other, which leads to the high complexity of model operations, and the model will only learn local noise when data are lost, STFGNN introduces data-driven temporal graphs to capture potential spatial-temporal dependencies, constructs predefined static topological graphs with more complete connections, and adopts parallel stacked spatial-temporal fusion layers and gated expansion convolutional fusion to achieve spatial correlation, non-neighborhood similarity, and temporal correlation in spatial-temporal networks modeling, with high prediction accuracy. However, for different specifications of road network structures, setting up temporal maps with fixed sparsity based on experience, insufficient or unnecessary connections can limit the prediction performance of the model.

**Table 2:** Comparison of prediction accuracy between MFSTAPFGCN and the baseline models.

Models	15 mins			30 mins			45 mins			60 mins		
	MAE	MAPE(%)	RMSE	MAE	MAPE(%)	RMSE	MAE	MAPE(%)	RMSE	MAE	MAPE(%)	RMSE
ARIMA	28.85	30.47	44.47	30.13	32.42	47.23	31.32	35.13	50.02	38.36	40.03	58.65
SVR	23.83	26.41	39.84	24.94	28.15	41.85	28.91	31.12	45.13	33.45	35.15	51.15
FC-LSTM	21.51	23.51	35.29	22.97	24.89	36.51	26.14	27.82	40.59	28.98	30.76	44.94
DCRNN	18.93	19.66	31.03	20.36	20.99	32.56	23.7	24.15	37.12	24.3	25.06	38.03
STGCN	18.37	19.4	30.34	19.32	20.51	31.98	21.74	22.83	34.65	22.38	23.51	35.61
ASTGCN	18.09	17.75	30.12	19.11	18.68	31.15	21.51	21.02	33.55	21.95	21.42	33.86
Graph WaveNet	19.85	19.71	32.94	20.93	20.69	33.82	24.45	24.26	38.74	24.97	25.81	39.33
STSGCN	17.83	17.13	29.56	18.39	17.68	30.12	20.19	19.52	32.65	21.36	20.7	33.48
STFGNN	16.88	16.41	28.45	17.41	16.98	28.99	18.83	18.42	30.88	20.74	20.39	31.93
STFAGN	16.24	15.5	28.26	17.18	16.42	28.62	18.52	17.78	30.31	20.03	19.43	31.51
**Our Model**	**15.15**	**14.06**	**26.83**	**16.83**	**15.86**	**27.64**	**17.53**	**16.82**	**28.92**	**18.84**	**18.15**	**30.17**

Based on STFGNN, STFAGN is designed for traffic prediction by combining fused convolutional layers with a new adaptive dependency matrix to handle the complex correlation, node heterogeneity of traffic flow data. In addition, STFAGN ignores the correlation and periodicity of each traffic state of the expressway. MFSTAPFGCN model, based on STFAGN, assigns different importance to the traffic state data of the road network at different moments with a multi-featured temporal attention mechanism, and models the daily periodicity similarity of the traffic flow data through splicing, dimensional expansion, and diffusion convolution operations. MFSTAPFGCN consistently and overwhelmingly outperforms all benchmark models in predicting traffic flow for the next 15 mins, improving the prediction accuracy by 6.7%, 9.3%, and 5%, respectively, over the accuracy-leading baseline model STFAGN.

The prediction accuracy of each model gradually decreases as the prediction time step increases, which indicates that long-term prediction of traffic flow is more difficult than short-term prediction. Among the STSGCN, STFGNN, STFAGN and MFSTAPFGCN models, STSGCN uses the local spatial-temporal graph mechanism to model the spatial-temporal correlation of traffic flow in short time synchronously, which overly emphasizes the local heterogeneous features and does not fully learn the global shared features in traffic flow, and does not fully deal with the long-term time dependence of traffic flow, and has the worst prediction results. STFGNN processes the potential non-proximity similarity in traffic flow data based on a data-driven time graph, and uses stacked gated diffusion convolution to model long-term time dependence, with slightly higher accuracy than STSGCN. STFAGN uses a fusion convolutional layer of adaptive dependency matrix to capture hidden spatial-temporal dependent traffic data, and the accuracy is slightly improved compared with STFGNN. In addition, STSGCN, STFGNN and STFAGN all ignore the correlation and periodicity of traffic states in expressways. The MFSTAPFGCN, which integrates the temporal correlation, spatial correlation, non-proximity correlation and periodic correlation of traffic flow data, is superior to other models in each evaluation index and each prediction time step.

### Ablation experiments

To further explore the role of different mechanisms in the MFSTAPFGCN model architecture in the prediction, we conducted relevant Ablation Experiments in predicting the traffic flow in the next 15 mins. Table 3 shows the experimental results of different mechanisms.

(1) STAPFGC: The multi-feature time attention mechanism is removed from the architecture of MFSTAPFGCN, and the other structures remain unchanged.
(2) MFSTAFGCN: The period processing module is removed from the architecture of MFSTAPFGCN, and other structures remain unchanged.
(3) MFSTPFGCN: The adaptive adjacency matrix is removed from the architecture of MFSTAPFGCN, and other structures remain unchanged.

**Table 3:** Ablation experiments of different structural components.

Model Elements	MAE	MAPE(%)	RMSE
STAPFGC	17.37	16.95	29.33
MFSTAFGCN	15.93	15.21	27.84
MFSTPFGCN	16.56	16.17	28.09
**MFSTAPFGCN**	**15.15**	**14.06**	**26.83**

**Fig 10:**
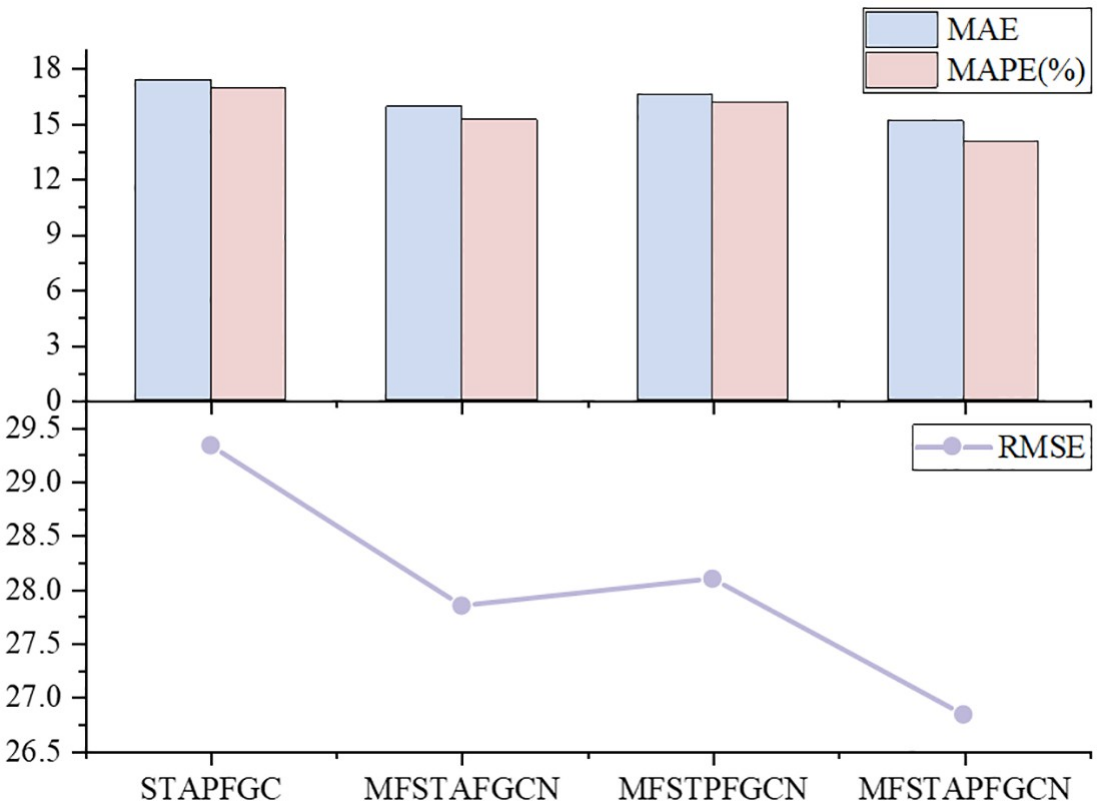
Comparison chart of performance indicators.

We can draw the following conclusions from Fig 10. The multi-featured temporal attention mechanism can provide more information to assist model prediction in the temporal dimension. The period processing layer can capture the daily similarity of traffic flow data and improve the model prediction accuracy. The adaptive adjacency matrix can model the road network structure more adequately and analyze data correlation more comprehensively, which can balance the limitation of dealing with node heterogeneity and improve the model prediction accuracy.

## Conclusion

In this paper, we propose an expressway traffic flow prediction model based on a multi-feature spatial-temporal adaptive periodic fusion graph convolutional network. In the temporal dimension, the relationship between different traffic variables at the same node in traffic forecasting is considered, and the dynamic temporal correlation between different times is captured using a multi-feature temporal attention mechanism. A spatial-temporal self-adaptive period fusion graph neural network is constructed using a period processing module, a spatial-temporal adaptive fusion graph module, and a gated convolution module, where the period processing module handles the period correlation and the learnable spatial-temporal fusion adjacency adaptively adjusts the spatial-temporal connections. The spatial-temporal adaptive fusion graph module is integrated with the gated convolution module to expand the reception on time series and stack them to comprehensively handle the spatial-temporal correlation of traffic flow data. The experiment results show that the model has some advantages in terms of mining features and prediction accuracy compared with existing models. The next research will collect data sets such as road function features, weather features, holidays, and social events to provide users with the most comprehensive information and form a complete intelligent traffic system, which is important for the subsequent development of smart expressways.

## Supporting information

S1 File ETC gantry transaction data. Desktop_ Lane_ In.csv is the ETC Gantry transaction data.
(CSV)

S2 File Road network topology data. t_etc_gantry.csv is the Road network topology data.
(CSV)
